# Expression of Concern: Study Protocols

**DOI:** 10.1097/MD.0000000000046330

**Published:** 2025-11-07

An Expression of Concern is being issued for the following articles.

Wang Y, Gong J. The effectiveness of intra-articular vs subacromial corticosteroid injection for frozen shoulder: Study protocol for a randomized controlled trial. Medicine (Baltimore). 2020 Apr;99(16):e19706. doi: 10.1097/MD.0000000000019706. PMID: 32311954; PMCID: PMC7440081.
Hu F, Chen X, Wu Y, Liu W. Prior knee arthroscopy effects on subsequent total knee arthroplasty: A protocol of match-controlled study. Medicine (Baltimore). 2020 Apr;99(17):e19844. doi: 10.1097/MD.0000000000019844. PMID: 32332637; PMCID: PMC7220770.
Yuan N, Shi J, Lin C, Li J. Adductor canal block versus periarticular infiltration for pain control following total knee arthroplasty: Study protocol for a randomized controlled trial. Medicine (Baltimore). 2020 Apr;99(17):e19903. doi: 10.1097/MD.0000000000019903. PMID: 32332669; PMCID: PMC7440221.
Tao Y, Mao Q, Wang J. Continuous versus single shot adductor canal block for postoperative pain relief after total knee arthroplasty: A protocol for randomized controlled trial. Medicine (Baltimore). 2020 Apr;99(17):e19918. doi: 10.1097/MD.0000000000019918. PMID: 32332672; PMCID: PMC7440233.
Zhao J, Luo WM, Li T. Extracorporeal shock wave therapy versus corticosteroid injection for chronic plantar fasciitis: A protocol of randomized controlled trial. Medicine (Baltimore). 2020 May;99(19):e19920. doi: 10.1097/MD.0000000000019920. PMID: 32384437; PMCID: PMC7220254.
Wang Q, Zhang Y, Du J, Lin X. Proximal versus distal adductor canal blocks for total knee arthroplasty: A protocol for randomized controlled trial. Medicine (Baltimore). 2020 May 29;99(22):e19995. doi: 10.1097/MD.0000000000019995. PMID: 32481369; PMCID: PMC12245286.
She GR, Zha ZG. Gap balancing versus measured resection in primary total knee arthroplasty: A retrospective cohort study protocol. Medicine (Baltimore). 2020 May;99(20):e20017. doi: 10.1097/MD.0000000000020017. PMID: 32443303; PMCID: PMC7253728.
Zhang Y, Xiong K, Li R, Yang L. Postoperative outcomes after total hip arthroplasty in patients with Parkinson disease: A protocol of case control study. Medicine (Baltimore). 2020 May;99(19):e20018. doi: 10.1097/MD.0000000000020018. PMID: 32384460; PMCID: PMC7220782.
Huang DG, Wu YL, Chen PF, Xia CL, Lin ZJ, Song JQ. Surgical or nonsurgical treatment for nontraumatic rotator cuff tears: Study protocol clinical trial. Medicine (Baltimore). 2020 May;99(18):e20027. doi: 10.1097/MD.0000000000020027. PMID: 32358381; PMCID: PMC7440173.
Li JQ, Hao-Xu, Sun ZG, Huang QS, Yao XD. The outcomes of post-traumatic arthritis vs osteoarthritis following primary total knee arthroplasty: A protocol of matched cohort study. Medicine (Baltimore). 2020 May;99(19):e20077. doi: 10.1097/MD.0000000000020077. PMID: 32384476; PMCID: PMC7220653.
Guo Y, Ma S, Wang J, Zhang Q, Wang S, Du Z. Cemented versus uncemented total knee arthroplasty in younger patients: A protocol of retrospective cohort trial. Medicine (Baltimore). 2020 May;99(18):e20087. doi: 10.1097/MD.0000000000020087. PMID: 32358393; PMCID: PMC7440262.
Zhang B, Sun S, Sheng B. Multiple versus single doses of dexamethasone in total hip arthroplasty: A protocol of randomized controlled trial. Medicine (Baltimore). 2020 May;99(19):e20147. doi: 10.1097/MD.0000000000020147. PMID: 32384500; PMCID: PMC7220463.
Ren Y, Liao J, Qin X, Yang J. Adductor canal block with periarticular infiltration versus periarticular infiltration alone after total knee arthroplasty: A randomized controlled trial protocol. Medicine (Baltimore). 2020 May;99(20):e20213. doi: 10.1097/MD.0000000000020213. PMID: 32443348; PMCID: PMC7254572.
Li WM, Li FD, Xu H, Sun LC. Analgesic impact of buprenorphine transdermal patch in total hip arthroplasty: A randomized controlled trial protocol. Medicine (Baltimore). 2020 Jun 12;99(24):e20405. doi: 10.1097/MD.0000000000020405. PMID: 32541462; PMCID: PMC7302595.
Wang S, Xu H, Ni W, Huang Q, Wang X. Unilateral versus bilateral balloon kyphoplasty in treatment of osteoporotic vertebral compression fractures: A randomized controlled trial protocol. Medicine (Baltimore). 2020 Jun 19;99(25):e20524. doi: 10.1097/MD.0000000000020524. PMID: 32569172; PMCID: PMC7310769.
Song F, Zheng Z. Intravenous versus topical tranexamic acid in lumbar interbody fusion: A protocol of randomized controlled trial. Medicine (Baltimore). 2020 Jun 12;99(24):e20619. doi: 10.1097/MD.0000000000020619. PMID: 32541498; PMCID: PMC7302681.
Chen J, Hu W, Li SM, Li XL, Yang ZM. The efficacy of ketamine in total knee arthroplasty: a randomized controlled trial protocol. Medicine (Baltimore). 2020 Jun 12;99(24):e20645. doi: 10.1097/MD.0000000000020645. PMID: 32541504; PMCID: PMC7302669.
Lin Y, Zhao J, Qiu H, Huang Y. All-inside versus inside-out suture techniques in arthroscopic meniscus repair: A prospective randomized study protocol. Medicine (Baltimore). 2020 Jul 2;99(27):e20688. doi: 10.1097/MD.0000000000020688. PMID: 32629640; PMCID: PMC7337569.
Wang LZ, Liu AH, Wen DJ, Zhou YT. Effects of prior anterior cruciate ligament reconstruction on clinical outcomes associated with total knee arthroplasty: A protocol of retrospective analysis. Medicine (Baltimore). 2020 Jun 19;99(25):e20767. doi: 10.1097/MD.0000000000020767. PMID: 32569220; PMCID: PMC7310844.
Wang D, Cang D, Wu Y, Wang S. Therapeutic effect of percutaneous vertebroplasty and nonoperative treatment on osteoporotic vertebral compression fracture: A randomized controlled trial protocol. Medicine (Baltimore). 2020 Jul 2;99(27):e20770. doi: 10.1097/MD.0000000000020770. PMID: 32629657; PMCID: PMC7337563.
Xue Q, Jiang W, Wang M, Sui J, Wang Y. Femoral nerve block vs adductor canal block after anterior cruciate ligament reconstruction under general anesthesia: A prospective randomized trial protocol. Medicine (Baltimore). 2020 Jul 10;99(28):e20776. doi: 10.1097/MD.0000000000020776. PMID: 32664070; PMCID: PMC7360326.
He J, Li Y. Randomized trial protocol of interscalene nerve block vs liposomal bupivacaine injection after total shoulder arthroplasty. Medicine (Baltimore). 2020 Jul 10;99(28):e20968. doi: 10.1097/MD.0000000000020968. PMID: 32664101; PMCID: PMC7360251.
Zhao Y, Yin K, Zhao H, Peng Z. Multiple screws versus sliding hip screws in femoral neck fractures: A protocol of cohort study. Medicine (Baltimore). 2020 Jul 2;99(27):e20970. doi: 10.1097/MD.0000000000020970. PMID: 32629708; PMCID: PMC7337602.
Lin Y, Zhao J, Qiu H, Huang Y. Comparison of arthroscopic single-row and double-row repair in the treatment of rotator cuff tears: A protocol of cohort analysis. Medicine (Baltimore). 2020 Jul 17;99(29):e21030. doi: 10.1097/MD.0000000000021030. PMID: 32702843; PMCID: PMC7373522.
Zhang Y, Wang X, Dong G. The analgesic efficiency of pregabalin for the treatment of postoperative pain in total hip arthroplasty: A randomized controlled study protocol. Medicine (Baltimore). 2020 Jul 2;99(27):e21071. doi: 10.1097/MD.0000000000021071. PMID: 32629738; PMCID: PMC7337400.
Cai J, Jiang W, Qiu B, Song Y. Efficacy and safety of epidural steroid injection following discectomy for patients with lumbar disc herniation: A protocol. Medicine (Baltimore). 2020 Jul 17;99(29):e21220. doi: 10.1097/MD.0000000000021220. PMID: 32702891; PMCID: PMC7373513.
Wu M, Li X, Li J, Chen Y. Operative vs conservative treatment in distal radius fractures: A protocol. Medicine (Baltimore). 2020 Jul 17;99(29):e21250. doi: 10.1097/MD.0000000000021250. PMID: 32702907; PMCID: PMC7373574.
Wu J, Guan T, Tian F, Liu X. Comparision of biportal endoscopic and microscopic decompression in treatment of lumbar spinal stenosis: A comparative study protocol. Medicine (Baltimore). 2020 Jul 24;99(30):e21309. doi: 10.1097/MD.0000000000021309. PMID: 32791717; PMCID: PMC7387062.
Zhou Y, Liu Z, Lei F, Xie K, Jia X. A randomized study protocol of microendoscopic versus open discectomy in treatment of lumbar disc herniation. Medicine (Baltimore). 2020 Jul 31;99(31):e21361. doi: 10.1097/MD.0000000000021361. PMID: 32756124; PMCID: PMC7402777.
Qiao HY, Xin L, Wu SL. Analgesic effect of extracorporeal shock-wave therapy for frozen shoulder: A randomized controlled trial protocol. Medicine (Baltimore). 2020 Jul 31;99(31):e21399. doi: 10.1097/MD.0000000000021399. PMID: 32756135; PMCID: PMC7402889.
Chen Y, Song H, Chen M, Xu H. The role of acupotomy in treatment of patients with lumbar spinal stenosis: A protocol for a randomized study. Medicine (Baltimore). 2020 Jul 31;99(31):e21444. doi: 10.1097/MD.0000000000021444. PMID: 32756160; PMCID: PMC7402714.
Yu Y, Sheng J, Zhou X. Computer-navigated versus conventional total knee arthroplasty: A randomized controlled trial protocol in China. Medicine (Baltimore). 2020 Aug 7;99(32):e21508. doi: 10.1097/MD.0000000000021508. PMID: 32769888; PMCID: PMC7593008.
Zheng H, Zhang Y, Wang H, Sun T, Sun Q. Comparison of perioperative hidden blood loss for intertrochanteric fractures in the elderly by different intramedullary fixations: A randomized controlled study protocol. Medicine (Baltimore). 2020 Nov 25;99(48):e21666. doi: 10.1097/MD.0000000000021666. PMID: 33235055; PMCID: PMC7710182.
Cong Z, Zhang L, Ma F. The efficacy and safety of the infiltration of the interspace between the popliteal artery and the capsule of the knee block in total knee arthroplasty: A prospective randomized trial protocol. Medicine (Baltimore). 2020 Aug 14;99(33):e21670. doi: 10.1097/MD.0000000000021670. PMID: 32872033; PMCID: PMC7437726.
Gao D, Liu X, Zhang F, Ding M. The efficacy and safety of multiple versus single doses dexamethasone in unicompartmental knee arthroplasty: A protocol of randomized controlled trial. Medicine (Baltimore). 2020 Aug 21;99(34):e21671. doi: 10.1097/MD.0000000000021671. PMID: 32846782; PMCID: PMC7447385.
Zhu L, Yang L, Wang Z, Cui H. Effects of adductor canal block on pain management compared with epidural analgesia for patients undergoing total knee arthroplasty: A randomized controlled trial protocol. Medicine (Baltimore). 2020 Aug 28;99(35):e21672. doi: 10.1097/MD.0000000000021672. PMID: 32871881; PMCID: PMC7458226.
Liu X, Jin G, Piao C, Zhong Z, Chang F, Huang B. Comparison of suture-button and screw fixation in the treatment of ankle syndesmotic injuries: Cohort study protocol. Medicine (Baltimore). 2020 Aug 7;99(32):e21679. doi: 10.1097/MD.0000000000021679. PMID: 32769937; PMCID: PMC7592993.
Xu H, Xia P, Zou X, Huang H. The efficacy and safety of intravenous tranexamic acid in anterior cruciate ligament reconstruction: A randomized controlled trial protocol. Medicine (Baltimore). 2020 Aug 21;99(34):e21747. doi: 10.1097/MD.0000000000021747. PMID: 32846798; PMCID: PMC7447492.
Xie X, Zhu J, Zhang H. Effects of extracorporeal shock wave therapy in patients with knee osteoarthritis: A cohort study protocol. Medicine (Baltimore). 2020 Aug 28;99(35):e21749. doi: 10.1097/MD.0000000000021749. PMID: 32871895; PMCID: PMC7458224.
Song X, Huang X, Yakufu M, Yan B, Feng C. Minimally invasive plate osteosynthesis or conventional intramedullary nailing for distal tibial fractures: A cohort study protocol. Medicine (Baltimore). 2020 Aug 14;99(33):e21779. doi: 10.1097/MD.0000000000021779. PMID: 32872079; PMCID: PMC7437767.
Li X, Guo D, Wang H, Zhou T. Effect and safety of intravenous versus oral acetaminophen after unicompartmental knee replacement: A protocol of randomized controlled study. Medicine (Baltimore). 2020 Aug 21;99(34):e21816. doi: 10.1097/MD.0000000000021816. PMID: 32846821; PMCID: PMC7447383.
Ye Z, Zhu W, Xi X, Wu Q. The efficacy of bidirectional barbed sutures for incision closure in total knee replacement: A protocol of randomized controlled trial. Medicine (Baltimore). 2020 Aug 21;99(34):e21867. doi: 10.1097/MD.0000000000021867. PMID: 32846841; PMCID: PMC7447360.
Yin Z, Qian P, Wu X, Gao F, Xu F. Unicondylar knee arthroplasty versus total knee arthroplasty in adults with isolated medial osteoarthritis: A matched study protocol. Medicine (Baltimore). 2020 Aug 28;99(35):e21868. doi: 10.1097/MD.0000000000021868. PMID: 32871911; PMCID: PMC7458234.
Zhang Q, Fan L. Comparison adductor canal block combined with local infiltration analgesia and adductor canal block alone for pain management after total knee arthroplasty: A randomized controlled trial protocol. Medicine (Baltimore). 2020 Aug 28;99(35):e21881. doi: 10.1097/MD.0000000000021881. PMID: 32871917; PMCID: PMC7458215.
Zhang W, Lin P, Zhang F, Wang J. Femoral nerve block versus obturator nerve block for pain management after total knee replacement: A randomized controlled trial protocol. Medicine (Baltimore). 2020 Sep 11;99(37):e21956. doi: 10.1097/MD.0000000000021956. PMID: 32925729; PMCID: PMC7489602.
Wang Y, Bai Y, Ma H, Wang S. Comparison of total disc arthroplasty and fusion in treatment of lumbar disc disease: A cohort study protocol. Medicine (Baltimore). 2020 Aug 28;99(35):e22024. doi: 10.1097/MD.0000000000022024. PMID: 32871957; PMCID: PMC7458242.
Liang D, Xue C, Liu W, Wang Y. What is the optimal regimen for intravenous dexamethasone administration in primary total hip arthroplasty?: A protocol of randomized controlled trial. Medicine (Baltimore). 2020 Sep 4;99(36):e22070. doi: 10.1097/MD.0000000000022070. PMID: 32899074; PMCID: PMC7478557.
Chen G, Gong M, Liu Y. Comparison of ropivacaine plus sufentanil and ropivacaine plus dexmedetomidine for labor epidural analgesia: A randomized controlled trial protocol. Medicine (Baltimore). 2020 Sep 4;99(36):e22113. doi: 10.1097/MD.0000000000022113. PMID: 32899094; PMCID: PMC7478759.
Tong Y, Jia X, Zhou Y, Feng D, Yuan D. Cervical arthroplasty versus anterior cervical discectomy in the treatment of symptomatic cervical spondylosis: A protocol. Medicine (Baltimore). 2020 Sep 11;99(37):e22145. doi: 10.1097/MD.0000000000022145. PMID: 32925770; PMCID: PMC7489585.
Song J, Qiao Y, Zhou Q, Zhang X. Fascia iliaca compartment block for analgesia in total hip replacement: A randomized controlled study protocol. Medicine (Baltimore). 2020 Sep 11;99(37):e22158. doi: 10.1097/MD.0000000000022158. PMID: 32925776; PMCID: PMC7489678.
Wang H, Ma L, Chen Y. Analgesic effects of low-dose ketamine after spinal fusion in adults: A protocol of prospective randomized trial. Medicine (Baltimore). 2020 Sep 18;99(38):e22162. doi: 10.1097/MD.0000000000022162. PMID: 32957340; PMCID: PMC7505386.
Wang L, Li P, Kou J, Hu C. The effect of previous acetabular fractures on total hip arthroplasty outcomes: A matched-controlled study protocol. Medicine (Baltimore). 2020 Sep 18;99(38):e22210. doi: 10.1097/MD.0000000000022210. PMID: 32957355; PMCID: PMC7505324.
Fu S, Wang Q, Fan C, Jiang Y. The efficacy of nursing intervention to reduce preoperative anxiety in patients with total knee arthroplasty: A protocol of prospective randomized trial. Medicine (Baltimore). 2020 Sep 18;99(38):e22213. doi: 10.1097/MD.0000000000022213. PMID: 32957356; PMCID: PMC7505384.
Yan X, Wang S. The efficacy of niacin supplementation in type 2 diabetes patients: Study protocol of a randomized controlled trial. Medicine (Baltimore). 2021 Mar 26;100(12):e22272. doi: 10.1097/MD.0000000000022272. PMID: 33761625; PMCID: PMC9282106.
Xu Y, Wang H, Yang M. Preoperative nursing visit reduces preoperative anxiety and postoperative complications in patients with laparoscopic cholecystectomy: A randomized clinical trial protocol. Medicine (Baltimore). 2020 Sep 18;99(38):e22314. doi: 10.1097/MD.0000000000022314. PMID: 32957397; PMCID: PMC7505285.
Wu L, Zou Y. Psychological nursing intervention reduces psychological distress in patients with thyroid cancer: A randomized clinical trial protocol. Medicine (Baltimore). 2020 Sep 18;99(38):e22346. doi: 10.1097/MD.0000000000022346. PMID: 32957406; PMCID: PMC7505398.
Chang Y, Zhou F, Fei L, Wang Z. The effect of preoperative degenerative spondylolisthesis on postoperative outcomes of degenerative lumbar spinal stenosis: A single-center cohort study protocol. Medicine (Baltimore). 2020 Nov 6;99(45):e22355. doi: 10.1097/MD.0000000000022355. PMID: 33157913; PMCID: PMC7647626.
Qi Z, Guo A, Ma L, Li Z, Yang B, Zhang J. Perioperative analgesia after intrathecal morphine or local infiltration anesthesia for total knee replacement: A protocol for randomized controlled trial. Medicine (Baltimore). 2020 Sep 25;99(39):e22394. doi: 10.1097/MD.0000000000022394. PMID: 32991462; PMCID: PMC7523756.
Qin B, Cui L, Ren Y, Zhang H. Retrospective cohort trial protocol of screw fixation compared with hemiarthroplasty for displaced femoral neck fractures in elderly patients. Medicine (Baltimore). 2020 Sep 25;99(39):e22397. doi: 10.1097/MD.0000000000022397. PMID: 32991464; PMCID: PMC7523755.
Huang Y, Gao D. The effectiveness of high intensity laser therapy in the patients with lumbar disc herniation: A protocol of randomized placebo-controlled trial. Medicine (Baltimore). 2020 Oct 9;99(41):e22520. doi: 10.1097/MD.0000000000022520. PMID: 33031293; PMCID: PMC7544293.
Yin D, Hui Y, Yang C, Xu Y. Effects of dapagliflozin on cardiovascular outcomes in type 2 diabetes: Study protocol of a randomized controlled trial. Medicine (Baltimore). 2020 Oct 9;99(41):e22660. doi: 10.1097/MD.0000000000022660. PMID: 33031329; PMCID: PMC7544418.
Sang X, Shan H, Hu J, Wu M. The efficacy of bilateral intervertebral foramen block for pain management in percutaneous endoscopic lumbar discectomy: A protocol for randomized controlled trial. Medicine (Baltimore). 2020 Oct 9;99(41):e22693. doi: 10.1097/MD.0000000000022693. PMID: 33031340; PMCID: PMC7544323.
Chen W, Zheng Y, Liang G, Chen G, Hu Y. Clinical effects of transforaminal approach vs interlaminar approach in treating lumbar disc herniation: A clinical study protocol. Medicine (Baltimore). 2020 Oct 30;99(44):e22701. doi: 10.1097/MD.0000000000022701. PMID: 33126307; PMCID: PMC7598806.
Hu Y, Zheng Y, Chen G, Chen W. Comparison of percutaneous endoscopic discectomy and microendoscopic discectomy in treatment of symptomatic lumbar disc herniation: A protocol of cohort study. Medicine (Baltimore). 2020 Oct 16;99(42):e22709. doi: 10.1097/MD.0000000000022709. PMID: 33080722; PMCID: PMC7572032.
Kong L, Chen L, Sun L, Tian X. Direct anterior approach or posterior approach in total hip arthroplasty: A direct comparative study protocol. Medicine (Baltimore). 2020 Oct 16;99(42):e22717. doi: 10.1097/MD.0000000000022717. PMID: 33080726; PMCID: PMC7571991.
Zheng Y, Wang H, Wang H, Xu J, Chen P. The efficacy of a phone assistance nursing program for functional outcomes in patients after shoulder instability surgery: A protocol for randomized controlled trial. Medicine (Baltimore). 2020 Oct 23;99(43):e22756. doi: 10.1097/MD.0000000000022756. PMID: 33120779; PMCID: PMC7581063.
Qi R, Li B, Xie T, Yin H. Surgical versus conservative management of fifth metatarsal fractures in adults: A protocol of retrospective study. Medicine (Baltimore). 2020 Oct 16;99(42):e22800. doi: 10.1097/MD.0000000000022800. PMID: 33080753; PMCID: PMC7571944.
Ye X, Wang F, Zeng W, Ding Y, Lv B. Comparison of empirical high-dose and low-dose of meropenem in critically ill patients with sepsis and septic shock: A randomized controlled study protocol. Medicine (Baltimore). 2020 Dec 18;99(51):e22829. doi: 10.1097/MD.0000000000022829. PMID: 33371058; PMCID: PMC7748186.
Zhu G, Wu C, Shen X. Rapid rehabilitation nursing improves clinical outcomes in postoperative patients with colorectal carcinoma: A protocol for randomized controlled trial. Medicine (Baltimore). 2020 Nov 6;99(45):e22857. doi: 10.1097/MD.0000000000022857. PMID: 33157927; PMCID: PMC7647534.
Zhang Z, Bai J, Huang Y, Wang L. Implementation of a clinical nursing pathway for percutaneous coronary intervention: A randomized controlled trial protocol. Medicine (Baltimore). 2020 Oct 23;99(43):e22866. doi: 10.1097/MD.0000000000022866. PMID: 33120826; PMCID: PMC7581146.
Yi P, Tang X, Yang F, Tan M. A retrospective controlled study protocol of transforaminal lumbar interbody fusion compared with posterior lumbar interbody fusion for spondylolisthesis. Medicine (Baltimore). 2020 Oct 30;99(44):e22878. doi: 10.1097/MD.0000000000022878. PMID: 33126336; PMCID: PMC7598866.
Song H, Wang M, Du H, Mu W. Comparison of locking plates and intramedullary nails in treatment of three-part or four-part proximal humeral neck fractures in elderly population: A randomized trial protocol. Medicine (Baltimore). 2020 Nov 13;99(46):e22914. doi: 10.1097/MD.0000000000022914. PMID: 33181658; PMCID: PMC7668495.
Fu S, Han H, Fan C, Jiang Y. Clinical nursing pathway improves the nursing satisfaction in patients with acute cerebral hemorrhage: A randomized controlled trial protocol. Medicine (Baltimore). 2020 Oct 30;99(44):e22989. doi: 10.1097/MD.0000000000022989. PMID: 33126374; PMCID: PMC7598808.
Gao X, Wan S, Lv J, Cheng W, Zhang Y. Comparison anterior minimally invasive oblique retroperitoneal approach and posterior transpedicular approach for debridement fusion in patients with lumbar vertebral osteomyelitis: A randomized controlled trial protocol. Medicine (Baltimore). 2020 Oct 30;99(44):e22990. doi: 10.1097/MD.0000000000022990. PMID: 33126375; PMCID: PMC7598824.
Wang D, Wang S, Lu K, Sun Y. Comparison of kinesio taping and sham taping in patients with chronic low back pain: A protocol of randomized controlled trial. Medicine (Baltimore). 2020 Nov 20;99(47):e23042. doi: 10.1097/MD.0000000000023042. PMID: 33217805; PMCID: PMC7676582.
Bo Y, Qin Y, Zang Y, Yang H. The suitable fixation for unstable intertrochanteric fractures: A protocol of comparative clinical study. Medicine (Baltimore). 2020 Oct 30;99(44):e23046. doi: 10.1097/MD.0000000000023046. PMID: 33126396; PMCID: PMC7598872.
Huang S, Yang Y, Yu X, Wang Y. The efficacy of gelatin sponge combined with moist wound-healing nursing intervention for the treatment of pressure ulcers: A randomized controlled trial protocol. Medicine (Baltimore). 2020 Nov 6;99(45):e23079. doi: 10.1097/MD.0000000000023079. PMID: 33157974; PMCID: PMC7647604.
Yang L, Ling D, Ye L, Zeng M. Psychological nursing intervention on anxiety and depression in patients with urinary incontinence after radical prostatectomy: A randomized controlled study protocol. Medicine (Baltimore). 2020 Nov 25;99(48):e23127. doi: 10.1097/MD.0000000000023127. PMID: 33235072; PMCID: PMC7710194.
Sun N, Li Y, Nie P. Standardized nursing and clinical efficacy of OxyContin in reducing oral mucosal pain in patients with nasopharyngeal carcinoma: A randomized, double-blind, placebo-controlled study protocol. Medicine (Baltimore). 2020 Dec 4;99(49):e23205. doi: 10.1097/MD.0000000000023205. PMID: 33285695; PMCID: PMC7717733.
Zhang F, Yang Y, Zhang H, Luo X. A comparative study protocol of external fixation versus volar plate in treating distal radius fracture. Medicine (Baltimore). 2020 Dec 11;99(50):e23231. doi: 10.1097/MD.0000000000023231. PMID: 33327243; PMCID: PMC7738129.
Xue Y, Lu T, Xu Y, Cao X. The efficacy of platelet-rich plasma in arthroscopic rotator cuff repair: A protocol of randomized controlled trial. Medicine (Baltimore). 2020 Dec 4;99(49):e23232. doi: 10.1097/MD.0000000000023232. PMID: 33285699; PMCID: PMC7717821.
Liu M, Shi B. Nursing intervention reduces depression for patients with rheumatoid arthritis: A randomized controlled trial protocol. Medicine (Baltimore). 2020 Nov 20;99(47):e23268. doi: 10.1097/MD.0000000000023268. PMID: 33217854; PMCID: PMC7676538.
Yin Q, Wang C, Yu J, Zhang Q. Quantitative assessment-based nursing intervention improves bowel function in patients with neurogenic bowel dysfunction after spinal cord injury: Study protocol for a randomized controlled study. Medicine (Baltimore). 2020 Dec 18;99(51):e23354. doi: 10.1097/MD.0000000000023354. PMID: 33371066; PMCID: PMC7748302.
Wang W, Xu F, Luo J, Zhu L. Conservative versus surgical treatment for Garden I hip fracture: A randomized controlled trial protocol. Medicine (Baltimore). 2020 Dec 24;99(52):e23378. doi: 10.1097/MD.0000000000023378. PMID: 33350726; PMCID: PMC7769300.
Zhang Z, Bai J, Huang Y. The efficacy of a nursing care and follow-up program for patients with heart failure: Study protocol for a randomized controlled trial. Medicine (Baltimore). 2020 Dec 4;99(49):e23380. doi: 10.1097/MD.0000000000023380. PMID: 33285722; PMCID: PMC7717929.
Ji X, Ding H. The efficacy of enteral nutrition combined with accelerated rehabilitation in non-small cell lung cancer surgery: A randomized controlled trial protocol. Medicine (Baltimore). 2020 Nov 25;99(48):e23382. doi: 10.1097/MD.0000000000023382. PMID: 33235112; PMCID: PMC7710191.
Gui Q, Su X, Lu Z, He J. Comparison between minimally invasive percutaneously and open pedicle screw fixation of thoracolumbar fracture: Prospective comparative study protocol. Medicine (Baltimore). 2020 Dec 4;99(49):e23403. doi: 10.1097/MD.0000000000023403. PMID: 33285728; PMCID: PMC7717750.
Yu H, Tan R, Lou B, Xue D. Efficacy of autologous platelet-rich plasma use for arthroscopic meniscal repair: A randomized trial protocol. Medicine (Baltimore). 2020 Nov 25;99(48):e23422. doi: 10.1097/MD.0000000000023422. PMID: 33235122; PMCID: PMC7710189.
Yin Y, Zhang X, Zhang K, He X. Unicompartmental knee replacement and high tibial osteotomy for medial unicompartmental knee osteoarthritis: A comparative study protocol. Medicine (Baltimore). 2020 Dec 4;99(49):e23454. doi: 10.1097/MD.0000000000023454. PMID: 33285742; PMCID: PMC7717739.
Li X, Du G, Liu W, Wang F. Music intervention improves the physical and mental status for patients with breast cancer: A protocol of randomized controlled trial. Medicine (Baltimore). 2020 Dec 4;99(49):e23461. doi: 10.1097/MD.0000000000023461. PMID: 33285746; PMCID: PMC7717794.
Zhao N, Yin F, Wu X, Zhong Y. The effectiveness of a WeChat-based multimodal nursing program for women with breast cancer: A randomized controlled trial protocol. Medicine (Baltimore). 2020 Dec 24;99(52):e23526. doi: 10.1097/MD.0000000000023526. PMID: 33350732; PMCID: PMC7769314.
Peng L, Liu H, Hu X, Liu J. Hemiarthroplasty versus total hip arthroplasty for displaced femoral neck fracture in patients older than 80 years: A randomized trial protocol. Medicine (Baltimore). 2020 Dec 11;99(50):e23530. doi: 10.1097/MD.0000000000023530. PMID: 33327300; PMCID: PMC7738070.
Peng L, Gao Y, Lu R, Zhou R. Efficacy of Omaha system-based nursing management on nutritional status in patients undergoing peritoneal dialysis: A randomized controlled trial protocol. Medicine (Baltimore). 2020 Dec 18;99(51):e23572. doi: 10.1097/MD.0000000000023572. PMID: 33371086; PMCID: PMC7748208.
Wang J, Li W. Self-care education program improves quality of life in patients with chronic heart failure: A randomized controlled study protocol. Medicine (Baltimore). 2020 Dec 11;99(50):e23621. doi: 10.1097/MD.0000000000023621. PMID: 33327336; PMCID: PMC7738048.
Huang L, Guo H, Xiu L, Wang H. Efficacy of individualized education in patients with type 2 diabetes mellitus: A randomized clinical study protocol. Medicine (Baltimore). 2020 Dec 11;99(50):e23625. doi: 10.1097/MD.0000000000023625. PMID: 33327339; PMCID: PMC7738066.
Li X, Qin K, Yuan C, Song S. The effect of enhancing quality of life in patients intervention for advanced lung cancer: Protocol for a randomized clinical study. Medicine (Baltimore). 2020 Dec 18;99(51):e23682. doi: 10.1097/MD.0000000000023682. PMID: 33371108; PMCID: PMC7748172.
Liao W, He X, Du Z, Long Y. Is a tourniquet necessary in arthroscopic anterior cruciate ligament reconstruction?: A randomized controlled study protocol. Medicine (Baltimore). 2021 Feb 5;100(5):e23724. doi: 10.1097/MD.0000000000023724. PMID: 33592830; PMCID: PMC7870236.
Liu P, Yao J, Qiu C. Nursing intervention using healing touch in total knee replacement: A randomized controlled study protocol. Medicine (Baltimore). 2021 Jan 22;100(3):e23735. doi: 10.1097/MD.0000000000023735. PMID: 33545940; PMCID: PMC7837904.
He ZX, Lu ZH, Ou J, Wu ZL. The role of bone grafts in displaced intra-articular calcaneal fractures: A prospective study protocol. Medicine (Baltimore). 2020 Dec 24;99(52):e23740. doi: 10.1097/MD.0000000000023740. PMID: 33350756; PMCID: PMC7769338.
Deng J, Wei K, Li M, Wang X, Tang Q. Paravertebral block reduces pain in elderly patients with percutaneous nephrolithotomy: A randomized controlled study protocol. Medicine (Baltimore). 2020 Dec 18;99(51):e23761. doi: 10.1097/MD.0000000000023761. PMID: 33371139; PMCID: PMC7748350.
Xue D, Lou B, Tan R, Yu H. Comparison between open reduction and internal fixation and minimally invasive surgery in management of Sanders type II calcaneal fracture: A randomized controlled trial protocol. Medicine (Baltimore). 2020 Dec 18;99(51):e23813. doi: 10.1097/MD.0000000000023813. PMID: 33371160; PMCID: PMC7748367.
Zhou C, Wang L. Corticosteroids vs autologous blood injection for lateral epicondylitis: Study protocol for a cohort trial. Medicine (Baltimore). 2020 Dec 18;99(51):e23842. doi: 10.1097/MD.0000000000023842. PMID: 33371166; PMCID: PMC7748333.
Song C, Li X, Ning X, Song S. Nursing case management for people with hypertension: A randomized controlled trial protocol. Medicine (Baltimore). 2020 Dec 24;99(52):e23850. doi: 10.1097/MD.0000000000023850. PMID: 33350776; PMCID: PMC7769350.
Miao B, Sun Y, Gong L, Liu W. Modular transitional nursing intervention improves pain-related self-management for cancer patients: Study protocol for a randomized controlled trial. Medicine (Baltimore). 2020 Dec 18;99(51):e23867. doi: 10.1097/MD.0000000000023867. PMID: 33371172; PMCID: PMC7748349.
Li D, Wang Y, Shen Y. A randomized study protocol comparing the platelet-rich plasma with hyaluronic acid in the treatment of symptomatic knee osteoarthritis. Medicine (Baltimore). 2021 Jan 15;100(2):e23881. doi: 10.1097/MD.0000000000023881. PMID: 33466131; PMCID: PMC7808492.
Chen J, Wang X. The efficacy and safety of local infiltration analgesia vs femoral nerve block after anterior cruciate ligament reconstruction: A retrospective trial protocol. Medicine (Baltimore). 2021 Jan 22;100(3):e23895. doi: 10.1097/MD.0000000000023895. PMID: 33545958; PMCID: PMC7837884.
Li G, Liao J, Su W. Open reduction and plate fixation versus sling in treatment of mid-shaft fractures of clavicle: A prospective randomized study protocol. Medicine (Baltimore). 2021 Jan 29;100(4):e23910. doi: 10.1097/MD.0000000000023910. PMID: 33530190; PMCID: PMC7850673.
Liang Y, Meng H, Li R, Yang J, Jia J, Hou Y. A randomized controlled trial protocol of the cardiovascular safety and efficacy of liraglutide in the treatment of type 2 diabetes. Medicine (Baltimore). 2021 Jan 22;100(3):e23948. doi: 10.1097/MD.0000000000023948. PMID: 33545972; PMCID: PMC7837848.
Zhang Y, Yang Y, Xiong G, Yu F. Long-term effects of regular exercise during pregnancy on overweight and obese gravidas: A protocol of randomized controlled trial. Medicine (Baltimore). 2021 Feb 5;100(5):e23955. doi: 10.1097/MD.0000000000023955. PMID: 33592849; PMCID: PMC7870188.
Chen Y, Lu T, Jiang X, Huang X. The effectiveness of specialized nursing interventions for patients with Parkinson disease: A randomized controlled study protocol. Medicine (Baltimore). 2021 Jan 15;100(2):e23972. doi: 10.1097/MD.0000000000023972. PMID: 33466136; PMCID: PMC7808514.
Wang L, Wang X, Wang X. The effectiveness of enteral nutrition for patients with primary liver cancer: A randomized controlled study protocol. Medicine (Baltimore). 2021 Jan 22;100(3):e23973. doi: 10.1097/MD.0000000000023973. PMID: 33545982; PMCID: PMC7837949.
Guo X, Men F, Han X, Wang Z. The efficacy of continuous nursing care for patients with chronic obstructive pulmonary disease: A randomized controlled trial protocol. Medicine (Baltimore). 2021 Jan 15;100(2):e23974. doi: 10.1097/MD.0000000000023974. PMID: 33466137; PMCID: PMC7808547.
Yang Y, Mu A, Wang Y. Early path nursing improves neurological function recovery in patients with intracerebral hemorrhage: Protocol for a randomized controlled trial. Medicine (Baltimore). 2021 Jan 8;100(1):e24020. doi: 10.1097/MD.0000000000024020. PMID: 33429767; PMCID: PMC7793312.
Jin Y, Li J, Wu S, Zhou F. Comparison of polyurethane foam dressing and hydrocolloid dressing in patients with pressure ulcers: A randomized controlled trial protocol. Medicine (Baltimore). 2021 Jan 15;100(2):e24165. doi: 10.1097/MD.0000000000024165. PMID: 33466190; PMCID: PMC7808478.
Zhu J, Chen M, Pang Y, Li S. Impact of lifestyle education for type 2 diabetes mellitus: Protocol for a randomized controlled trial. Medicine (Baltimore). 2021 Jan 8;100(1):e24208. doi: 10.1097/MD.0000000000024208. PMID: 33429812; PMCID: PMC7793339.
Xia W, Liu T, Lei J. The efficacy of a short one-on-one nursing intervention in people with coronary heart disease: A randomized controlled trial protocol. Medicine (Baltimore). 2021 Jan 29;100(4):e24405. doi: 10.1097/MD.0000000000024405. PMID: 33530238; PMCID: PMC7850645.
Zuo J, Pan X, Shen W. Low back pain: Influence of early MR imaging or CT on treatment and outcome - a randomized controlled study protocol. Medicine (Baltimore). 2021 Mar 5;100(9):e24535. doi: 10.1097/MD.0000000000024535. PMID: 33655920; PMCID: PMC7939158.
Li Y, Li L, Guo Z, Zhang S. Comparative effectiveness of furosemide vs torasemide in symptomatic therapy in heart failure patients: A randomized controlled study protocol. Medicine (Baltimore). 2021 Feb 19;100(7):e24661. doi: 10.1097/MD.0000000000024661. PMID: 33607802; PMCID: PMC7899842.
Lou X, Xu H. The effectiveness of an emergency department nursing intervention on psychological symptoms and self-care capacities: A randomized controlled study protocol. Medicine (Baltimore). 2021 May 28;100(21):e24763. doi: 10.1097/MD.0000000000024763. PMID: 34032691; PMCID: PMC8154481.
Li S, Yang M, Tang L, Zhou Y. The synergistic effects of applying low-level laser therapy plus ultrasound on pain and muscle function in patients with knee osteoarthritis: A protocol of a randomized double-blind study. Medicine (Baltimore). 2021 Mar 12;100(10):e24764. doi: 10.1097/MD.0000000000024764. PMID: 33725832; PMCID: PMC7969322.
Shan H, Jiao G, Cheng X, Dou Z. Safety and efficacy of edaravone for patients with acute stroke: A protocol for randomized clinical trial. Medicine (Baltimore). 2021 Feb 26;100(8):e24811. doi: 10.1097/MD.0000000000024811. PMID: 33663100; PMCID: PMC7909220.
Yuan Z, Jiao N, Liu X, Liu C. The effect of web-based educational intervention on psychological status and blood glucose in newly diagnosed patients with diabetes type 2 in rural China: A protocol for randomized trial. Medicine (Baltimore). 2021 Feb 26;100(8):e24937. doi: 10.1097/MD.0000000000024937. PMID: 33663132; PMCID: PMC7909169.
Gu J, Dai B, Shi X, He Z. Clinical short-term outcomes of articular-sided and bursal-sided partial-thickness rotator cuff tears of less than 50% in a single surgeon series: A protocol of randomized controlled trial. Medicine (Baltimore). 2021 Mar 12;100(10):e24965. doi: 10.1097/MD.0000000000024965. PMID: 33725860; PMCID: PMC7969290.
Zhang J, Diao P, Zhang L. Intravenous versus oral omeprazole on patients with high risk bleeding peptic ulcers: A prospective randomized clinical trial protocol. Medicine (Baltimore). 2021 Apr 9;100(14):e25136. doi: 10.1097/MD.0000000000025136. PMID: 33832076; PMCID: PMC8036073.
Xie H, Tang Y, Cheng F, Chu J. The efficacy of combined music therapy and Tai Chi for major depressive disorder: Study protocol for a randomized controlled trial. Medicine (Baltimore). 2021 Mar 26;100(12):e25241. doi: 10.1097/MD.0000000000025241. PMID: 33761717; PMCID: PMC9281998.
Zeng R, Tian K, Xiao Z. Effectiveness of thoracic kinesio taping on respiratory function and muscle strength in patients with chronic obstructive pulmonary disease: A protocol of randomized, double-blind placebo-controlled trial. Medicine (Baltimore). 2021 Apr 9;100(14):e25269. doi: 10.1097/MD.0000000000025269. PMID: 33832089; PMCID: PMC8036067.

After publication, concerns were raised regarding the authorship and the validity of the protocol registration.

An investigation is being conducted in line with the Committee on Publication Ethics (COPE) guidelines and Wolters Kluwer’s policies.

The Expression of Concern will remain in place until the investigation has been completed. If the editors and publisher can reach a conclusion, they will take any action that is deemed necessary. If they have determined that they cannot reach a satisfactory conclusion with the information available to them, a further notification will be published to update the research community.

